# The impact of chronic disease management on primary care doctors in Switzerland: a qualitative study

**DOI:** 10.1186/s12875-018-0833-3

**Published:** 2018-09-11

**Authors:** Olivia Braillard, Anbreen Slama-Chaudhry, Catherine Joly, Nicolas Perone, David Beran

**Affiliations:** 1 Department of Community Medicine, Primary and Emergency Care, Geneva University Hospitals, 1205 Geneva, Switzerland; 2 Department of Community Health and Care, Geneva University Hospitals, 1205 Geneva, Switzerland; 3 Division of Tropical and Humanitarian Medicine, Geneva University Hospitals and University of Geneva, 1205 Geneva, Switzerland

**Keywords:** Primary health care, General practice, Chronic disease, Multimorbidity, Time management, Qualitative research

## Abstract

**Background:**

Patient-centeredness and therapeutic relationship are widely explored as a means to address the challenge of chronic disease and multi-morbidity management, however research focusing on the perspective of doctors is still rare. In this study, we aimed to explore the impact of the patient’s chronic disease(s) on their healthcare provider.

**Methods:**

A qualitative approach was taken using semi-structured interviews with general practitioners working in outpatient clinics either in individual practices or in a hospital setting in Geneva, Switzerland. Codes were developed through an iterative process and using grounded theory an inductive coding scheme was performed to identify the key themes. Throughout the analysis process the research team reviewed the analysis and refined the coding scheme.

**Results:**

Twenty interviews, 10 in each practice type, allowed for saturation to be reached. The following themes relevant to the impact of managing chronic diseases emerge around the issue of feeling powerless as a doctor; facing the patient’s socio-economic context; guidelines versus the reality of the patient; time; and taking on the patient’s burden. Primary care practitioners face an emotional burden linked with their powerlessness and work conditions, but also with the empathetic bond with their patients and their circumstances. Doctors seem poorly prepared for this emotional strain. The health system is also not facilitating this with time constraints and guidelines unsuitable for the patient’s reality.

**Conclusions:**

Chronic disease and multi-morbidity management is a challenge for healthcare providers. This has its roots in patient characteristics, the overall health system and healthcare providers themselves. Structural changes need to be implemented at different levels: medical education; health systems; adapted guidelines; leading to an overall environment that favors the development of the therapeutic relationship.

**Electronic supplementary material:**

The online version of this article (10.1186/s12875-018-0833-3) contains supplementary material, which is available to authorized users.

## Background

Chronic diseases are defined by their long duration and slow progression with the current challenge for health systems not only in managing the individual chronic disease, but most notably multi-morbid individuals [1]. Chronic diseases and multi-morbidity lead to both financial and organizational burdens on the health system [2–5]. Patients with multiple chronic diseases face greater healthcare utilization and costs, decreased self-reported health status, depression and reduced functional capacity [1]. In addition the challenge of polypharmacy and managing multiple conditions, including possibly mental health issues, is both a challenge for the individual and healthcare provider(s) [6]. In the United States 84% of total health care costs are related to chronic disease [7] and in the United Kingdom a retrospective cohort study found that 78% of consultations at primary health care are for people with more than one chronic condition [8].

Very little data exists on the burden of different chronic diseases in Switzerland [9]. In a study of individuals with insurance from a specific company aged 65 or older from all of Switzerland it was found that 76.6% were multi-morbid [10]. Compared to non-multi-morbid individuals these individuals had on average 15.7 consultations versus 4.4 and their associated costs were 5.5 times higher. In Switzerland models for the management of chronic disease are not as well established as in other high income settings^11^ with barriers to effectively implement chronic care linked to the organization of the health system, its financing and weaknesses at primary health care level. This means that comprehensive models that have been developed elsewhere may not be implemented in the same way in Switzerland [11, 12].

Given these limitations for primary care doctors in the Swiss health system, and that very little focus and research on the impact of the patient’s chronic disease(s) on primary care doctors exists [13], the aim of this study is to explore the impact of the patient’s chronic disease(s) on their healthcare provider.

## Methods

### Context

In Switzerland, due to the federal system, each of the 26 cantons is responsible for the provision of health services, financing of public hospitals as well as subsidizing some of the population’s insurance premiums [14]. The Federal government provides the legislative framework which regulates the insurance market, defines the healthcare services covered by the basic insurance package and the way in which these are paid for. One-third of health care spending in Switzerland is from out of pocket payments [14].

Income for primary care doctors’ in outpatient settings is dependent on the number of patients they see and the technical acts they perform. The government has delegated to an association of Swiss insurance companies the task of “economically evaluating” doctors by comparing costs generated by each practitioner to an average. Hospital based practitioners do not have the same financial pressure, however time per patient is an issue.

This study took place in Geneva, one of the 26 Swiss cantons [15]. It is characterized by a very diverse population (total 494,000), high density of doctors and the largest university teaching hospital of Switzerland.

### Methodological approach

A qualitative approach was taken, with an interview guide created by the team of investigators including three Primary Care specialists (OB, ASC, NP), 1 Public Health Research specialist with a PhD (DB), and a Nurse specialized in Chronic Disease management and Patient Education (CJ). All contributors have experience in qualitative research projects. Discussion topics were related to Chronic Care Model (CCM) [16] and these were used as grand tour questions [17]. The CCM provides a framework for the necessary components to provide integrated chronic disease management. It comprises not only the role of the health system and healthcare provider, but also such elements as policies, the community and patient. For the purpose of this study the CCM was used as a framework to build the interview guide (cf Additional file 1) to guide the interview through the different levels of care (patient, doctor, health system) and the domains that could influence the quality of chronic care (Resources and policies, organization of health care, self-management support, decision support, delivery system design, clinical information systems).

The protocol received ethical approval from the Geneva University Hospitals (HUG) Research Ethics Committee (reference number 14–022). All participants signed a written informed consent form and it was made clear to them they could withdraw from the study at any time. Convenience sampling was used to gain a wide diversity of views. Participants were recruited through an e-mail announcement informing every all primary care practitioners in Geneva (570 in private practices and 40 in the Division of Primary Care Medicine at the HUG) about the study. Selection was done based on those who answered first.

**Fig. 1:**
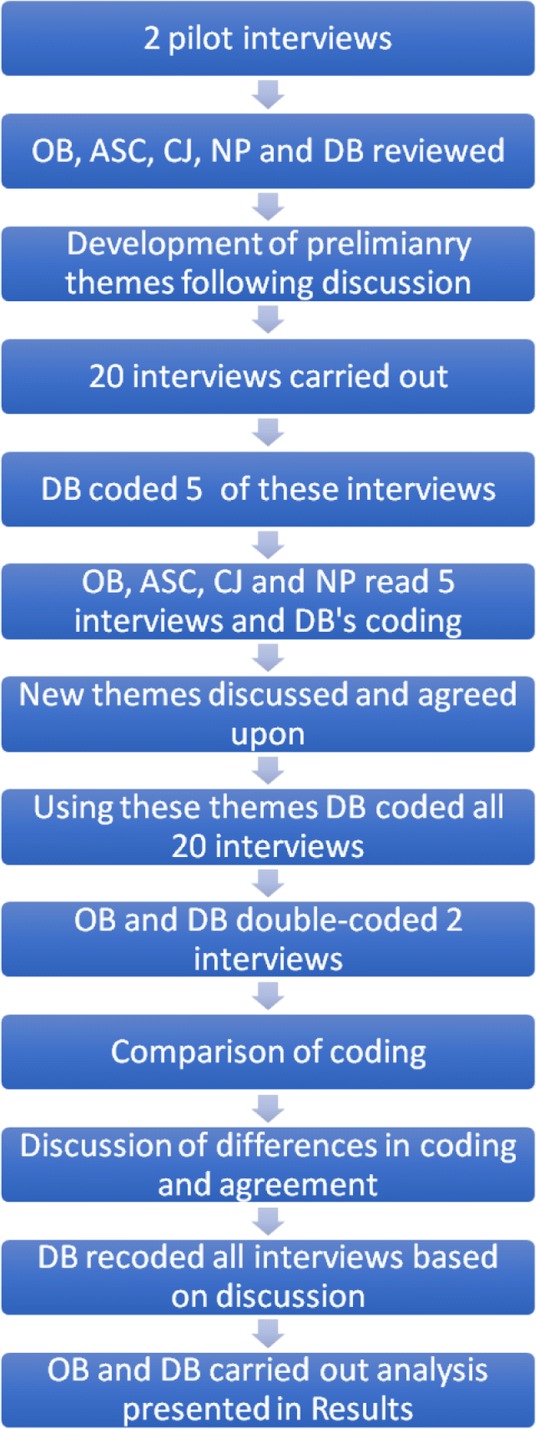
Data analysis process

The researcher (CJ) conducting interviews was experienced in interview techniques. She had no professional relation with participants. All participants were made aware of the general objectives of the research which was to explore practitioners’ difficulties, needs and resources in the caring for patients with chronic diseases. Interviews were digitally recorded and transcribed verbatim by a person unrelated to the research team and were not returned to the interviewees for comment. Throughout this process, all data were anonymized to guarantee confidentiality using unique codes comprised of SMPR and a number based on the order of interview. These codes are used to present the quotes from interviewees in the results. Interviews took place at participants’ workplace (HUG or private practices) between July and August 2014. Only the participant and the interviewer (CJ) were present. Participants received a CHF 50 voucher after completion of interview, in compensation for their time. Interviews were carried out until theoretical saturation was achieved. All interviews were audio recorded and transcribed in French. No field notes were taken. The research and analysis process is presented in Fig. 1.

**Fig. 2:**
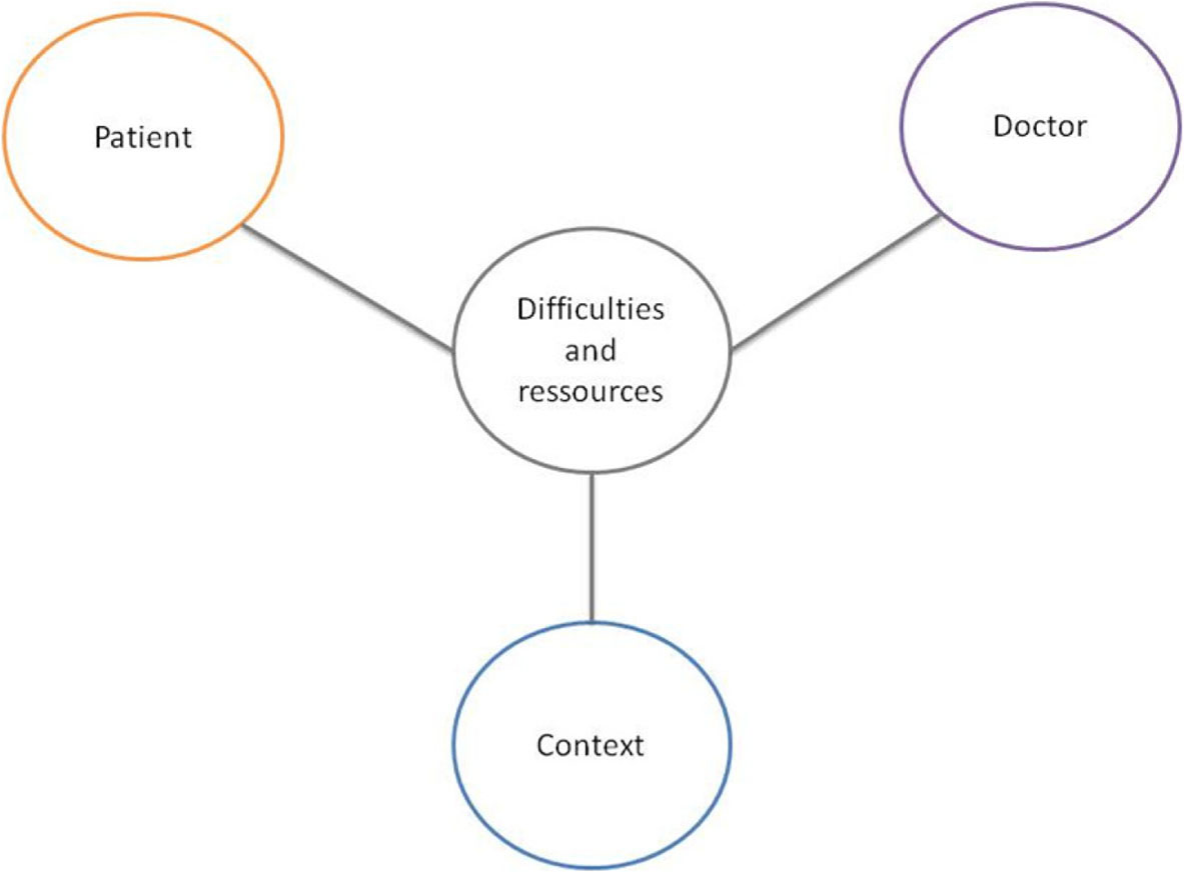
Visual tool used to conduct interviews

The interview guide was tested on 2 interviews (excluded from analysis) to check its relevance and clarity. After the 2 test interviews, the guide was refined and a visual tool to support the interviews used. (Fig. 2) It was felt that a visual tool was necessary following the 2 test interviews to guide the discussion and allow for an interaction around the key issues between the interviewer and interviewee. The research team read and analyzed 2 test interviews in order to create a coding scheme. These codes were triangulated among the research team to achieve a consensus on their validity for analysis. During the interviews notes and comments were added to Fig. 2 to highlight key issues, remind the interviewer and/or interviewee of some points or as a means to return to certain key issues.

A grounded theory analysis was used. Grounded Theory provides a systematic framework for collecting and analysing qualitative data that is flexible and assists in the creation of theories “grounded in the data collected” [18–21]. An inductive coding scheme was used and analysis was performed using NVIVO 11 for Mac (NVivo qualitative data analysis Software; QSR International Pty Ltd). Analyses were carried out in French, and then translated in English for publication purposes. Five interviews were then analyzed by one member of the team (DB) using this initial coding scheme. Additional codes were added and defined during this process. The research team then reviewed the analysis and refined the coding scheme. Two interviews were double coded (OB and DB) using the second coding scheme and the research team discussed any discrepancy in the analysis. All interviews were then re-coded (DB) using the last coding scheme. Analysis and coding was then discussed and validated by the team. Throughout this process, disagreements and discrepancies were discussed among the researchers until an agreement was validated by the whole team.

## Results

Twenty interviews, 10 in each practice type, allowed for saturation to be reached. All participants completed their interviews and there were no repeat interviews. Mean duration of interviews was 51 min (minimum 24 min, maximum 65 min). The characteristics of the interviewees are detailed in Table 1.

**Table 1:** Characteristics of interviewees

Code	Practice type	Age	Years of practice
SMPR1	Private practice	52	25
SMPR2	Private practice	59	34
SMPR3	Private practice	55	25
SMPR4	Private practice	53	30
SMPR5	Private practice	65	30
SMPR6	Private practice	67	40
SMPR7	Private practice	58	34
SMPR8	Private practice	42	18
SMPR9	Private practice	67	32
SMPR 10	Private practice	40	21
SMPR 20	Hospital	44	17
SMPR 21	Hospital	53	29
SMPR 22	Hospital	34	7
SMPR 23	Hospital	42	13
SMPR 24	Hospital	35	5
SMPR 25	Hospital	53	27
SMPR 26	Hospital	34	7
SMPR 27	Hospital	39	12
SMPR 28	Hospital	36	10
SMPR 29	Hospital	41	16
	Average Overall (Range)	48 (34–67)	22 (5–40)

The following themes relevant to the impact of managing chronic diseases emerge around the issue of feeling powerless as a doctor; facing the patient’s reality; guidelines versus the reality of the patient; time; and taking on the patient’s burden.

### Feeling powerless as a doctor

Many of those interviewed expressed feeling powerless. SMPR3 states this as “when you are a young doctor, you like to be the savior.” SMPR28 describes how for infectious diseases and broken arms doctors can easily find solutions, but that for chronic diseases “it is almost like we give them medicines and we make them sick.” SMPR10 and SMPR24 highlight the challenge of patients coming back with recurring complaints that they are unable to provide a solution for.

This powerlessness was also fueled by the perceived patient’s view as expressed by SMPR26 “the patient does not necessarily expect to get better and that is difficult to accept as a doctor”. SMPR 10 adds “if we feel that the intervention we are proposing will not change anything our feeling powerless as a doctor increases.” This is complemented by SMPR23 saying, “I do not cure them! I just provide treatment!” with SMPR2 highlighting how a transition is needed in the view of being a doctor as a savior and being able to see the limits of what a doctor can do. SMPR3 adds, “With time we are able to relativize a lot and redefine our role as a partner and not a healer.”

### The patients’ reality

Some of this feeling powerless was linked to varying patient characteristics such as social, psychiatric and disease factors. SMPR20 describes this as “the limit of my action in the limits of the context [of the patient] and the difficulty of adapting what I am saying to a reality that I do not know well, therefore the feeling that sometimes I am not in sync with what the patient is living.” Different elements comprise the patient’s reality, not only including the disease(s) that the doctor is managing, but also their socio-economic characteristics. Patient related factors impacted the management of the patient’s chronic disease and could be divided into disease related factors and multi-morbidity and the patient’s social context.

#### Disease related factors and multi-morbidity

With disease related factors, different challenges present themselves at distinctive stages of the disease process. For example, at the time of diagnosis “For some the announcement of having diabetes all of a sudden is an upheaval, we are going to tell them that they need to do various things and I realize that this is a bomb. We put bombs for these patients, but for us it is just diabetes.” (SMPR24) This “bomb” at the time of diagnosis is followed by challenges throughout the management of the disease until the last stage of the disease, when the doctor needs to explain “if you are no longer able to breathe it is because you have smoked for 40 years and your body is letting go […] they have to understand that they are going to die.” (SMPR23).

End of life care was identified as a situation with additional challenges (SMPR23), as were chronic disease management in geriatric populations (SMPR23), mental health issues (SMPR7), and specific diseases e.g. HIV/AIDS, diabetes (SMPR7). SMPR24 also describes a vicious cycle in that chronic disease can be disabling for the individual therefore impacting the individual’s mental state, which in turn impacts management. As stated by SMPR24, “Somatic chronic diseases often have consequences or come in parallel to difficult psychological situations and it is then difficult to identify what is the cause and consequence.” Many doctors in discussing multi-morbidity focused more on psychiatric diseases and addiction (SMPR4, SMPR7, SMPR23, SMPR24 and SMPR25) rather than somatic conditions. SMPR4 portrayed multi-morbidity as “We are juggling many eggs at the same time.” This practitioner gave the example of one of their patients having alcohol problems after a bypass surgery and how to manage this added challenge in an individual who already had an eating disorder, problems with their body image, depression and other chronic diseases. The challenge was also how to manage all these issues in parallel (SMPR20).

#### Patient’s social context

A variety of social factors impacting the management of the patient’s chronic disease are described by the interviewees. “There are some contexts where as a doctor I take a blood pressure, I use my stethoscope, but that is not the problem! Patients just need to eat, be washed and then access care”(SMPR23). SMPR2, SMPR21, SMPR23, SMPR24 and SMPR25 add to this complexity in mentioning the importance of the patient’s surroundings, including: work, life events, family context, financial means, cultural factors, seclusion, and even illiteracy. SMPR25 summarizes this as “the problem is much more non-medical: it is really the surroundings, work, life events that have a bigger impact than us.” A specific example is SMPR1 describing how a patient with dyspnea may also be losing his job, having marital trouble and these factors accumulate as elements in their overall suffering.

Doctors seemed ill equipped to manage the patient’s social context linked to cultural issues (SMPR5, SMPR7, SMPR20, SMPR21, SMPR23 and SMPR26) and precarious financial situations (SMPR2, SMPR3 and SMPR20). Cultural issues related to barriers in effective communication with the patient and therefore patient’s understanding of for example taking medicines. Financial issues were related to the fact that some patients could not access care they needed (SMPR2), lacked financial means to exercise or afford healthy food (SMPR20), or due to job constraints did not see management of their condition as a priority (SMPR20).

### Guidelines versus reality

Guidelines which support evidence-based medicine goals are focused on individual diseases and do not take account the complexity of the patients’ reality including socio-economic factors. Therefore, the doctor’s efforts to follow guidelines are often a failure. SMPR3 describes a patient who could not check his blood glucose twice a day before and after meals because of his work schedule. This shows that the patients’ reality cannot be controlled by the physician. SMPR22 expresses this as “there are things that we can change in the context, and things for which we aren’t there. We talk about theory, but once at home...”

This “theory” refers to not being able to follow guidelines due to the nature of the patients disease(s) and other related factors, adding to the doctor’s feeling of powerlessness and frustration. Participants described how they are able to put into perspective the importance of guidelines, but that these are limited as guidelines are, “only statistical considerations” (SMPR3), whereas they need to deal with individuals with varying needs.

SMPR20 describes his approach as “trying to find openings where they are, by trying to find strategies which are literally adapted to patient’s reality. [It’s] useless to talk about changing diet, when the patient eats at a soup kitchen”. SMPR24 summarizes it with this statement: “with chronic patients, you need to see further than guidelines, see patient’s resources, understand his story, see how he lives with his illness [..]. There are many facets you can’t set aside”.

### Dealing with complexity requires time

Time was a recurrent theme presented by the doctors interviewed. Interviewees described how they struggled with all the tasks they should perform within a limited time frame of a consultation. SMPR28 lists each task he’s supposed to do during a single consultation only for diabetes and adds, “And all this in 30 minutes”. Therefore, doctors have to prioritize, “in which order and at what time we do things” (SMPR 29), with the risk of “los[ing] track of which exams to do” (SMPR26) or omit something such as stopping a specific medicine no longer required (SMPR 29).

Besides identified specific tasks, many interviewees stated that more time is required to create a therapeutic relationship. For SMPR20, this relationship is “built with the duration and by knowing people” and it is necessary to “enter into patient’s life” (SMPR29). SMPR20 summarizes this need for time to build relationship as “if we don’t have time, we don’t take care of a person but of a disease”.

Due to the limit of time imposed by Swiss health system (through its insurance and funding system), many doctors felt frustrated. Several interviewees were quite spirited when discussing this as SMPR2 said, “I can only do my job as a doctor if you allow me more time”. SMPR7 had received a warning from an insurance company about the time spent with his patients and stated, “I told them that a society which didn’t take time for the bond was a dying one, and that in no way I would agree to reduce the time of my consultations”.

### Impact of taking on the patient’s burden

Many of the doctors interviewed took on their patient’s burden with them feeling for their patient and their situation. This had a negative impact in them requiring a lot of energy and a emotional impact. In addition, this also had a positive impact finding motivation and pleasure in some interactions.

Some interviewees stated how consuming it was to take on the patient’s burden, and how it made them feel alone. SMPR7 stated “we are bearer of everything […] psychological, familial issues, the despair, the advancing age”. SMPR24 compares some patients as a “very heavy stone to pull”, SMPR23 confessed feeling “like pedaling in vain” and being exhausted.

SMPR24 describes the “intensity with the chronic patient that we don’t have with any patient [..] it impacts us strongly when in a given day we see patients who feel down because no treatment is working, we still have to continue to accompany them, not to let them down.” Due to this bond built on the therapeutic relationship, several interviewees described being affected by their patients’ situations (SMPR1, SMPR7, SMPR10, SMPR23 and SMPR24). This is described in different ways, such as “to feel sadness” (SMPR7) about their situation, their issues, or even “to feel what they feel” (SMPR1). All these feelings resulted in energy used to “digest” these emotions (SMPR10).

Interviewees also described positive impact resulting from their work with chronic patients. When SMPR2 talks about a resilient 88-year-old patient with many issues, he relates the pleasure he has to see his fighting spirit, and how it also helps him as a doctor keep motivated. Interviewees described this phenomenon when the patient took their advice (SMPR26), showed resilience facing serious issues (SMPR2, SMPR10), the long-term relationship (SMPR28) or simply through the stories shared by the patients (SMPR2, SMPR10). SMPR10 summarizes, “It’s wonderful this relation we can have with people sharing the story of their life, their beliefs […] It’s really touching but in the same time, it also takes energy”.

## Discussion

### Summary

This study describes the complexity from doctors’ perspective of managing individuals with chronic diseases. The CCM provided a useful framework for the interview guide as it enabled the investigation of the key element of the doctor/patient interactions. This relationship between patient and healthcare provider is central to this model and essential for the management of chronic diseases. Although wider community and policy issues were not assessed these appeared as barriers to the management of individuals with chronic diseases, such as time limitations on consultations and wider social factors impacting health.

### Strengths and limitations

The aim of this study was exploratory in nature therefore a qualitative approach was adopted. As with any qualitative study sampling, data collection, analysis and presentation, as well as contextual bias are limitations [22]. These were mitigated by the experience and diversity of the team involved in this research as well as a clear description of the methods included. To the authors’ knowledge this study is unique in its approach and findings and thus serves as a contribution to the literature in understanding the various challenges that primary care doctors face in providing care to patients with chronic diseases.

### Comparison with existing literature

Most literature on the issue of primary health care and chronic disease are disease or patient-centered and there are few studies describing doctor’s perspective [23–26]. Research shows that 20–40% of primary care doctors are emotionally exhausted due to work-related factors: their income linked to number of patients seen, for especially healthcare providers in private practice, hours of work and stress [27]. A systematic review on primary healthcare provider’s perspective on multi-morbidity found that their reaction facing multi-morbidity could reach “something close to despair” [28]. Kenning’s study [29] shows the emotional strain experienced by practitioners with the management of complex patients who show little improvement or willingness to engage in their own care. Our study is unique in that it reveals that the emotional burden faced by primary care practitioners is not only linked to their powerlessness and work conditions, but also to the empathetic bond with their patients and their circumstances.

The doctor’s feeling of powerlessness is a key finding from this study. Factors such as the training of health professionals, the way guidelines are developed with a disease focus, organization of the health system all contribute to this. From a health system perspective, these components can all be changed. However, patient factors such as disease related factors, multi-morbidity and their social context cannot be modified. It is for the health system and doctor to adapt to these in order to find the appropriate responses. The main challenge identified were the time constraints imposed by the organization of the health system thus preventing the establishment of a therapeutic relationship. The therapeutic relationship is broadly acknowledged as the cornerstone of chronic disease management [29], yet doctors in our study seem poorly prepared for this emotional strain with health system factors also not facilitating this.

Guidelines which should help doctors do not take into account patients’ reality and complexity. Parekh and al [30]. in their proposal to improve the management of multi-morbidity include a goal on equipping clinicians. However, this focuses on training, guidelines and identifying best practice and tools. These recommendations fail to address the complexity of the patient, the impact of this on the healthcare provider and the limitations the health system imposes.

### Implications for research and practice

Health systems need to drastically change how they support both care for chronic patients and the impact this has on doctors. Wagner et al. [31] point out that each element of the health system, including policies, need to change in order to enable effective care for chronic patients. Most health systems do not provide the propitious environment for patient-centered care or effective teamwork [32]. Although there is some experience in implementing the CCM in practice this is only as part of studies, pilots, or in specific contexts [33]. Financial issues need to be addressed in that time limits on consultations with complex patients are a short-term saving [34]. Resources are also needed to truly have integrated care including inter-professional teams to address the multiple needs of the patients and alleviate the doctor’s workload. This should include care coordination as a cornerstone of this effective team and be led by either the primary care practitioner or a trained case manager [35–37]. Prevention must become a top-priority [38] and must be financially rewarded [39]. Well-being and resilience promotion [40, 41] should be a part of medical training to build emotional coping abilities [42]. As management of multi-morbidity involves uncertainty, which is badly tolerated by medical students, and can be a reason not to become a primary care doctor, medical students and young doctors should be taught to cope with this [43]. All of these elements need to be delivered at primary care level and as SMPR28 concludes regarding the primary care specialty: “for nothing else in the world I would do another specialty […] but it’s not easy”. Health systems need to find ways to care for their caregivers, such as SMPR28 in order to ensure proper care is provided to chronic and multi-morbid patients.

## Conclusion

Chronic disease and multi-morbidity management is a challenge for healthcare providers. This has its roots in patient characteristics, the overall health system and healthcare providers themselves. Structural changes need to be implemented at different levels: medical education; health systems; adapted guidelines; leading to an overall environment that favors the development of the therapeutic relationship. This therapeutic relationship is a cornerstone for properly managing complex patients. To have this requires an investment in terms of time, energy and emotion, but health systems currently do not provide the enabling environment for this. Structural changes need to be implemented at different levels: medical education needs to prepare doctors for this emotional strain; health systems need to find innovative financing mechanisms; consultations need to be adapted and move towards team-based integrated care; and tools such as adapted guidelines need to be developed and used.

## Additional file

Additional file 1: Interview guide. (DOCX 20 kb)
