# Supplementary material for: The impact of chronic disease management on primary care doctors in Switzerland: a qualitative study
Source: BMC Fam Pract. 2018 Sep 11;19:159. doi: 10.1186/s12875-018-0833-3 (PMC6134721; doi:10.1186/s12875-018-0833-3)
Supplement: Supplementary file 1 — Interview guide. (DOCX 20 kb) [file 12875_2018_833_MOESM1_ESM.docx]

**Interview guide**

Translation of the first version, 11.03.2014

**INTRODUCTION : The framework of the interview**

**Duration: 1 hour**

**Purpose:** To conduct a qualitative study to explore aspects of the current management of chronic disease patients by primary care physicians in the city and at Geneva University Hospitals.

**Frame:** in the physician's office

**Content:** three phases: difficulties experienced/identified in follow-up, resources, strategies used in follow-up for chronic patients, expectations/needs to improve their follow-up.

**Confidentiality:** Recorded anonymous interviews, then transcribed for qualitative analysis. A unique code will be used to identify each interview by the person transcribing the interviews. This person will be different from the person who will conduct the interviews so the confidentiality of the doctors will be ensured in this way. Also during the transcription any mention of a person by name will be replaced by a Mr/Mrs/Doctor X.

**Feedback:** Depending on your interest, the result of this study will be sent to you.

**QUESTIONING :**

**1- What difficulties do you encounter in the follow-up of your patients suffering from chronic diseases? (30min)**

About :

1. Organization of health care

2. Patient self-management support (or empowerment of the patient and family)

3. Clinical decision support

4. The clinical information system

5. Use of community resources

6. The overall organization of the health system (reimbursement of benefits, access to specialized services)

Related to :

- The patient
- The context
- Yourself

**2-What strategies have you already implemented or are you thinking of implementing to facilitate/improve the follow-up of your chronically ill patients? (15min)**

Community (family involvement...)

The health system (network with IMAD, specialists, etc)

Services (medical file, offer of care)

In your own skills

The skills of patients and their families

**3- To improve the management of patients suffering from chronic diseases, what would be your expectations/needs? (10min)**

For yourself

For your chronic patients

Other

**CONCLUSION :**

Acknowledgement

Ask if you wish to be informed of what's next?
